# Louhi 2010: Special issue on Text and Data Mining of Health Documents

**DOI:** 10.1186/2041-1480-2-S3-I1

**Published:** 2011-07-14

**Authors:** Hercules Dalianis, Martin Hassel, Sumithra Velupillai

**Affiliations:** 1 Department of Computer and System Sciences, (DSV) Stockholm University Forum 100, 164 40 Kista, Sweden

## Abstract

The papers presented in this supplement focus and reflect on computer use in every-day clinical work in hospitals and clinics such as electronic health record systems, pre-processing for computer aided summaries, clinical coding, computer decision systems, as well as related ethical concerns and security. Much of this work concerns itself by necessity with incorporation and development of language processing tools and methods, and as such this supplement aims at providing an arena for reporting on development in a diversity of languages. In the supplement we can read about some of the challenges identified above.

## Introduction

An increasing production of clinical textual data from electronic health record systems has pushed on the development of new methods and approaches for text and data mining. Since the nature of clinical data is complex and relate to many sources of human knowledge it is a challenging task that requires several approaches. Clinical text is noisy with numerous spelling errors and non-standard abbreviations, as well as incomplete sentences [1]. In parallel, people working in both patient care as well as biomedical research and education has discovered the potential of the combination of this abundant source of clinical data and advanced tools. One example is the automatic indexing of clinical findings, observations, diseases and treatments that is of high relevance for clinical work and research in general. The indexed data needs to be retrieved and this poses new challenges in the clinical and medical domain in which there is a lot of implicit and tacit knowledge.

Implementing decision support and guidelines to reach evidence-based practice is a specific area of interest [2]. Detecting adverse events is another field that needs a high-quality system to detect findings and events in clinical documents [3]. In several other areas, information retrieval and access of clinical text are of outmost importance – from everyday clinical work to translational biomedical research. Meystre et al. [4] present an elaborate and extensive overview of the research area. Health care consumer Web sites as well as news Web sites contain important information worthwhile monitoring to extract both information about specific diseases directed to laymen as well as epidemiological information. The papers presented in this supplement aim at exploring computational methods and tools to improve and support the work in these different fields. This supplement focuses and reflects on computer use in every-day clinical work in hospitals and clinics such as electronic health record systems, pre-processing for computer aided summaries, clinical coding, computer decision systems, as well as related ethical concerns and security. Much of this work concerns itself by necessity with incorporation and development of language processing tools and methods, and as such this supplement aims at providing an arena for reporting on development in a diversity of languages. In the supplement we can read about some of the challenges identified above.

The workshop that preceded the outcome of this supplement was the Second Louhi Workshop on Text and Data Mining of Health Documents, Louhi 2010, that took place in Los Angeles, California, USA in June 2010. For more information on the workshop, see http://dsv.su.se/en/louhi10/.

## Summary of selected papers

In the paper by Allvin et al. [5] the authors have carried out both a qualitative and quantitative comparative study of Finnish and Swedish nursing narratives from two intensive care units. As the Swedish and Finnish languages belong to different language groups, while the countries are culturally closely related, this study explores how this might influence what is expressed in the narratives. The conclusions are that the domain specific attributes as for example non-standard abbreviations, missing subject and spelling errors are similar over languages, but the electronic records systems differ in that the headings are defined by the nurses while writing in Finland but in Sweden they are predefined in templates.

The paper by Halgrim et al. [6] describes a hybrid system for medical extraction based on both rule based and statistical classifiers. The system is applied on English narrative clinical records from the i2b2 challenge and uses several rule based processing steps where field detection is one significant step detecting if a medication occurs in narrative text or in a list of medications. The medication is divided into six different sub classes as medication name, dosage, frequency, duration, mode and reason for the medication. The authors obtained comparable results to the top systems in the 2009 i2b2 challenge.

To interpret clinical text and specifically to detect when a diagnosis is affirmed, one needs to perform negation detection. Skeppstedt [7] has ported the negation detection system NegEx by Chapman et al. [8] which is written for English clinical text, to SwedishThe paper describes the porting process in detail and evaluates the Swedish version of NegEx on 558 manually classified sentences containing negation triggers. Even though the precision was lower for the Swedish NegEx than for the English NegEx, it could still be concluded that the same trigger phrase approach is viable in a Swedish context since many negated propositions were identified through a limited set of trigger phrases.

Building resources for information retrieval purposes with a clinical focus is very important for the research community. With these, it may be possible to understand different user perspectives and to develop methods for tailored requirements. Friberg Heppin [9] describes the Swedish MedEval, a test collection for information retrieval research, where assessments have been made both for topical relevance and target reader groups (physicians and patients). This collection has been used for experiments with different user scenarios. Moreover, the collection has two indexes, one containing split compounds, which is very useful for collections in morphologically complex languages such as Swedish.

Reliability, or trustworthiness, of information is also an important aspect in information retrieval for medical purposes. Martin [10] describes work on annotating Web documents containing health care information directed to health care consumers for reliability and type. Inter-rater reliability agreement results in this study are promising and opens up the possibility for automatic classification of medical information on the Web, which is very important for both professionals and lay people.

## Competing interests

The authors declare that they have no competing interests.

## Dedication

This supplement is dedicated to professor Hans Åhlfeldt, Linköping University, Sweden, a colleague and also reviewer of Louhi 2010 workshop that passed away suddenly in September 2010.
